# Mechanisms of enhanced cardiorespiratory performance under hyperoxia differ with exposure duration in yellowtail kingfish

**DOI:** 10.1098/rspb.2023.2557

**Published:** 2024-06-19

**Authors:** Daniel Morgenroth, Tristan McArley, Javed Khan, Erik Sandblom

**Affiliations:** ^1^ Department of Biological and Environmental Sciences, University of Gothenburg, PO Box 463, Gothenburg 405 30, Sweden; ^2^ Department of Animal Environment and Health, Swedish University of Agricultural Sciences, Gothenburg 405 30, Sweden; ^3^ National Institute of Water and Atmospheric Research, Northland Marine Research Centre, PO Box 147, Ruakaka 0151, New Zealand; ^4^ CH4 Global, 48 Greys Avenue, Auckland 1010, New Zealand

**Keywords:** hyperoxia, aerobic scope, cardiovascular function, tissue O_2_ extraction

## Abstract

Hyperoxia has been shown to expand the aerobic capacity of some fishes, although there have been very few studies examining the underlying mechanisms and how they vary across different exposure durations. Here, we investigated the cardiorespiratory function of yellowtail kingfish (*Seriola lalandi*) acutely (~20 h) and chronically (3–5 weeks) acclimated to hyperoxia (~200% air saturation). Our results show that the aerobic performance of kingfish is limited in normoxia and increases with environmental hyperoxia. The aerobic scope was elevated in both hyperoxia treatments driven by a ~33% increase in maximum O_2_ uptake (MO_2max_), although the mechanisms differed across treatments. Fish acutely transferred to hyperoxia primarily elevated tissue O_2_ extraction, while increased stroke volume-mediated maximum cardiac output was the main driving factor in chronically acclimated fish. Still, an improved O_2_ delivery to the heart in chronic hyperoxia was not the only explanatory factor as such. Here, maximum cardiac output only increased in chronic hyperoxia compared with normoxia when plastic ventricular growth occurred, as increased stroke volume was partly enabled by an ~8%–12% larger relative ventricular mass. Our findings suggest that hyperoxia may be used long term to boost cardiorespiratory function potentially rendering fish more resilient to metabolically challenging events and stages in their life cycle.

## 1. Introduction

Oxygen (O_2_) supersaturation (i.e. hyperoxia) is a common phenomenon in aquatic environments where photosynthetic organisms abound. It generally follows a diel pattern with photosynthetic rates increasing at sunrise and water O_2_ levels peaking around sunset. Since water temperature often follows a similar pattern, hyperoxia has been suggested to be of substantial importance for sustaining metabolic performance in many shallow-water organisms, as peak water O_2_ saturation roughly coincides with peak water temperatures, when routine O_2_ demand of most non-photosynthetic organisms is at its highest [1]⁠. This suggests that organisms may use waters rich in photosynthetic activity as a metabolic refuge during metabolically challenging periods (e.g. acute warming [1]⁠). Additionally, artificial hyperoxia achieved via O_2_ supplementation is a common practice in aquaculture, most commonly to improve water conditions and avoid sudden bouts of hypoxia, although sometimes because it is thought to improve some aspects of animal performance (e.g. growth [2]⁠). It is owing to this ecological relevance as well as potential benefits in aquaculture that efforts have intensified to unravel the physiological impacts of hyperoxia in fish [1–4]⁠.

Aerobic scope is defined as the capacity of an animal to elevate its aerobic metabolic rate (often approximated as O_2_ uptake, MO_2_) above maintenance levels and can be calculated as the difference between maximum O_2_ uptake (MO_2max_) and standard metabolic rate (SMR [5]⁠). In other words, aerobic scope represents the aerobic energy budget that can be allocated into functions beyond staying alive motionlessly in a non-absorptive state, including growth, reproduction, locomotion and immune function, which is why it has been proposed as a key physiological determinant of whole-animal performance and a measure of an animal’s fitness [6–8]⁠. Fish exposed either acutely or chronically to environmental hyperoxia often show an expanded aerobic scope through increases in MO_2max_, while SMR remains generally unaffected [9–14]⁠. The mechanisms that allow for an elevated MO_2max_ in hyperoxia, however, are not fully understood and have only been comprehensively explored in a limited number of fish species.

The mechanisms whereby overall tissue O_2_ delivery is maintained are summarized by the Fick equation: MO_2_ = cardiac output × arterial–venous O_2_ content difference, where cardiac output is the product of heart rate and stroke volume, and the arterial–venous O_2_ content difference is the difference between O_2_ content in arterial (CaO_2_) and venous blood and represents the amount of O_2_ extracted by the tissues per volume of blood. The elevated MO_2max_ of rainbow trout (*Oncorhynchus mykiss*) acutely exposed to hyperoxia is in part owing to improved cardiac capacity, as indicated by an increased cardiac output following exhaustive exercise under hyperoxia [9,10]⁠. Moreover, in both exhaustively exercised and acutely heated fish, the elevated cardiac output under hyperoxia appears to be achieved via enhanced cardiac contractility as stroke volume is elevated, while heart rate remains largely unaffected [9,15,16]⁠. Relevant in this regard is the fact that the majority of teleostean fishes have an exclusively spongy myocardium and rely on venous blood returning to the heart for oxygenation, while some species also have a compact myocardium perfused with well-oxygenated arterial blood by a dedicated coronary circulation [17]⁠. However, in both rainbow trout that possess coronaries and in European perch (*Perca fluviatilis*) that lack coronaries, the increased cardiac output and capacity to sustain a higher MO_2_ under hyperoxia are most evident at temperatures close to their critical thermal maximum [11,15,16]⁠. This coincides with an elevated venous partial pressure of O_2_ (PvO_2_), suggesting that the increased cardiac output that drives the higher MO_2_ in hyperoxia is largely possible thanks to enhanced cardiac oxygenation [15,16]⁠. Depending on the method employed to exert MO_2max_, arterial partial pressure of O_2_ (PaO_2_) and CaO_2_ may drop significantly immediately following exhaustive exercise [10,18,19]⁠. Hyperoxia allows for the maintenance of elevated post-exhaustive exercise PaO_2_ and CaO_2_, thus, the larger CaO_2_ following exercise allows for an increased arterial–venous O_2_ content difference, also contributing to the larger MO_2max_ under hyperoxia [10]⁠. Furthermore, in fish species with a coronary circulation, such as salmonids, the protective effects of hyperoxia on arterial oxygenation also mean that the coronary O_2_ delivery to the compact myocardium is elevated under hyperoxic conditions, likely contributing to improving cardiac function as well [10]⁠.

Commonly, most studies exploring the effects of hyperoxia on aerobic capacity of fish have been carried out following relatively short exposure times (≤22 h [2]⁠). While short exposures may reflect conditions experienced by wild fish in nature (e.g. daily fluctuations in O_2_ levels or when swimming through hyperoxic waters, etc.), they do not reflect the chronic hyperoxia experienced by some farmed fishes. To the best of our knowledge, the effects of prolonged sustained hyperoxia on aerobic capacity have only been explored in one fish species. Skeeles *et al*. [14]⁠ recently showed that the common galaxias (*Galaxias maculatus*) acutely exposed to hyperoxia display an MO_2max_-driven elevation in aerobic scope, which remained elevated following 5 months of acclimation to these conditions. Even so, an important question that remains to be addressed is whether the metabolic changes observed in fish chronically acclimated to hyperoxia are accompanied by cardiorespiratory changes resembling those in acutely exposed fish.

The yellowtail kingfish (*Seriola lalandi*) is a large marine pelagic fish found in subtropical and temperate waters across the Southern Hemisphere [20]⁠. It is an athletic swimmer with an active lifestyle, clearly reflected in its elevated SMR and aerobic scope [21,22]⁠. Owing to its robustness to environmental changes, fast growth, commercial value and feasibility to be bred and reared in captivity, kingfish have recently experienced a rapid expansion as an aquaculture species [23–25]⁠. As a result, there is considerable research interest in multiple performance aspects of this species, including the effects of environmental variables on its physiological responses and performance across life stages [21,26–28]⁠. Here, we investigated the effects of acute (~20 h) and chronic (>3 weeks) exposure to hyperoxia on the cardiorespiratory performance (MO_2_, cardiac output, heart rate, stroke volume and arterial–venous O_2_ content difference) of kingfish at rest and following an exhaustive stress protocol. We also analysed the effects of hyperoxia on excess post-exercise O_2_ consumption/uptake (EPOC), which can be defined as the O_2_ debt acquired during exhaustive exercise [29,30]⁠. We hypothesized that both acute and chronic hyperoxia would elevate MO_2max_ and aerobic scope, and tested whether the mechanisms differ with acclimation duration. More specifically, we tested whether plastic physiological changes along the O_2_ transport cascade occur as a result of prolonged exposure to hyperoxia. These included shifts in the relative importance of increased stroke volume-mediated cardiac output and arterial–venous O_2_ difference in improving respiratory performance. To complement these studies, we measured the haematological properties of yellowtail kingfish and analysed whether any changes in cardiac performance in hyperoxia were reflected in plastic morphological changes of the heart (e.g. ventricular growth).

## 2. Materials and methods

### (a) Experimental animals and rearing conditions

The yellowtail kingfish used in these experiments were F1 and F2 individuals derived from wild-caught broodstock and fully reared on-site in the National Institute of Water & Atmospheric Research Ltd. (NIWA) at the Northland Marine Research Centre (NMRC) in Ruakaka, New Zealand. All fish were Passive Integrated Transponder (PIT)-tagged in the abdominal muscles following light anaesthesia with 10 ppm isoeugenol (Aqui-S, New Zealand) and then divided into two acclimation conditions: hyperoxia (201 ± 3% air saturation) and normoxia (106 ± 1% air saturation). Fish were then maintained under these conditions at a biomass density of <10 kg m^−3^ in 1.5 m^3^ tanks at a temperature of ~24°C under an 18:6 h day:night photoperiod and fed a 1.5% body mass ratio with 6 mm pellets (Yellowtail 6P, EWOS, 48% protein, 20% oil, 0.8% fibre, 9.7% ash) twice daily. Water was UV sterilized, filtered to 20 μm and maintained at the desired experimental air saturation using an OxyGuard Pacific control platform (OxyGuard, Denmark), which consisted of a probe monitoring dissolved O_2_ and a control system that injected O_2_ automatically as needed to maintain the desired O_2_ levels. Salinity was checked daily using a handheld refractometer and remained stable at ~35 ppt throughout the experimental period. Additionally, to ensure good water quality, the total gas pressure (Handy Polaris probe, Oxyguard, Denmark), ammonia concentration (low range reagent sets, AmVer Test’N Tube, USA), pH (portable pH meter, Seven2Go Pro, Mettler Toledo, USA) and dissolved CO_2_ concentration (CO_2_ analyzer, Oxyguard, Denmark) were measured every 7–10 days and remained within normal limits throughout the experimental period. Fish were maintained at their respective acclimation conditions for 3–5 weeks before the cardiorespiratory experiments started. All experimental procedures were approved by NIWA’s Animal Ethics Committee (Application code: AEC237).

### (b) Surgical procedures

Food was withheld for at least 2 days before surgery. Fish were anaesthetized in water of their respective acclimation condition containing 150 mg l^−1^ of MS-222 (Tricaine methanesulfonate, Scanvacc, Hvam, Norway). Once opercular movements ceased, the body mass and fork length were recorded, and the fish was placed laterally on wet foam on a surgery table and a continuous flow of aerated water (~16°C) containing 75 mg l^−1^ of MS-222 was circulated across the gills. All fish were instrumented with a 2.5 mm Transonic PSL type (PS-Series Probe with lateral cable) transit-time blood flow probe (Transonic Systems, Ithaca, NY, USA) around the ventral aorta for measurements of cardiac output and heart rate. After surgery, fish were divided into three experimental treatment groups, each group consisting of 12 fish. Fish acclimated to hyperoxic conditions were maintained in hyperoxia throughout the duration of the experiment (Hyperoxia_chronic_). One group of fish acclimated to normoxic conditions was maintained in normoxia (Normoxia), while another group of fish acclimated to normoxia were acutely transferred to hyperoxic conditions after the surgery and remained in hyperoxia for the duration of the experiment (Hyperoxia_acute_). Fish were typically first maintained in holding tubes for >5 h to closely monitor post-surgical recovery and then transferred to one of six 10 l Perspex respirometers submerged in one of two 800 l experimental tanks, upon which continuous recordings of MO_2_, heart rate and cardiac output started. Each respirometer was covered with polyvinyl chloride (PVC) and each tank was surrounded with black plastic drapes to minimize visual disturbances.

### (c) Experimental protocol and sampling

In total, the fish were given 20 h of recovery (i.e. overnight) before subjecting them to an exhaustive exercise protocol. Briefly, the rear end of the respirometer was opened, allowing for the tail of the fish to be repeatedly grabbed and pinched, which elicited a vigorous swimming/struggle response that rendered the fish exhausted within 3 min. Within 20 s from the exhaustive exercise, cardiorespiratory recordings commenced. Pure O_2_ was generally bubbled into the tanks to ensure that air saturation remained at the desired levels within the respirometers. Cardiorespiratory variables were measured for up to 6 h after the exhaustive protocol or until cardiorespiratory variables had returned and stabilized at baseline levels, following which the fish were removed from the respirometers, anaesthetized with water containing 150 mg l^−1^ of MS-222, and euthanized via a sharp blow to the head. Blood was immediately obtained via caudal puncture (~1 ml) using heparinized syringes and pH was measured at 24°C using a two-point calibrated handheld pH meter (Sentron SI400, Sentron Europe, Leek, The Netherlands). Haemoglobin concentration ([haemoglobin]) was measured using a handheld Hb 201+ analyzer (Hemocue, Ängelholm, Sweden), and the values were adjusted for fish blood [31]⁠. Haematocrit was determined as the fraction of red blood cells in capillary tubes following 5 min of centrifugation at 10 000*g*. The spleen and heart ventricle were dissected out, blotted dry with tissue wipes and their weight was recorded. For the heart ventricle, this was done after careful removal of the atrium and bulbus arteriosus, as well as any remaining blood in the ventricle lumen. We then preserved the ventricle in 70% ethanol for further analyses of myocardial muscle composition (see the following sections).

### (d) Data acquisition and analytical procedures

We recorded the rate of whole-animal O_2_ uptake (MO_2_) using intermittent-flow respirometry where the per cent air saturation inside the respirometer was continuously measured using an O_2_ optode connected to a Firesting O_2_ system (PyroScience, Aachen, Germany) [32]⁠. Automated flush pumps (flow rate 20 l min^−1^, Eheim Universal 1200, Deizisau, Germany), were set to flush the respirometers for 5 min every 7 min (i.e. 2 min measurement cycles). The Transonic flow probes were connected to a Transonic 400 series blood flow meter (Transonic Systems, Ithaca, NY, USA). All probes were individually bench calibrated at 24°C following the same protocol as Morgenroth *et al*. [33]⁠ using a pulsatile pump (Model 1407 PBP, Harvard Apparatus, Holliston, MA, USA). Analogue output signals from the flow meter and O_2_ optode system were recorded at a sampling rate of 10 Hz using a PowerLab system (ADInstruments, Castle Hill, Australia) and LabChart pro data acquisition software (version 7.3.2, ADInstruments, Castle Hill, Australia).

MO_2_ was calculated from the slope of the decline in per cent air saturation between flushes using the following formula: MO_2_ = (*V*_r_ – *V*_f_) × (Δ%Sat/*t*) × *α*; where *V*_r_ is the volume of the respirometer, *V*_f_ is the volume of the fish assuming that 1 g of tissue equals 1 ml of water, Δ%Sat/*t* is the change in per cent O_2_ saturation per time and *α* is the temperature-, salinity- and atmospheric pressure-dependent solubility coefficient of O_2_ [5]. The first ~30 s of each measurement cycle was excluded from the slope determination to ensure the inclusion of only the linear section of the decline in O_2_. SMR was calculated as the mean of the lowest 20% of all MO_2_ values obtained throughout the whole 20+ h of recordings, with measurements two standard deviations below the mean of the lowest 20% removed as outliers. MO_2max_ was calculated as the highest MO_2_ value obtained at any point following exercise. The lowest 20% were chosen instead of the more commonly used lowest 10% to maximize the number of fish that achieve EPOC repayment for each treatment. Aerobic scope was then calculated as the difference between SMR and MO_2max_ [5]_⁠_. EPOC was calculated as the area between the MO_2_ curve following the stress protocol and SMR + 10% using GraphPad Prism 9.1.2 following the method of Zhang *et al*. [34]⁠. Briefly, before analysis, individual MO_2_ traces were smoothed by removing routine MO_2_ values that were 10% larger than the previous value. EPOC duration was defined as the time in hours between the exhaustive protocol and the intersection of the MO_2_ trace with the individual SMR + 10%. The rate of EPOC repayment was defined as EPOC/EPOC duration. We cleaned the respirometers thoroughly after each trial, and measured background respiration before and after each individual experiment and was negligible throughout the study (<0.2% of the MO_2_ slope).

Heart rate was calculated from the pulsating blood flow signal, and stroke volume was calculated as cardiac output/heart rate. The arterial–venous O_2_ difference was estimated as MO_2_/cardiac output (Fick’s principle’s equation). All cardiovascular variables were measured simultaneously with MO_2_ recordings, and cardiovascular variables measured concomitantly to SMR, MO_2max_ and aerobic scope are referred to henceforth as resting, maximum and scope. Additionally, cardiorespiratory dynamics were assessed immediately prior to and at six time points following the exhaustive protocol and thus comprised: pre-exhaustion values (average of the two last cycles prior to exhaustive protocol), immediately after the exhaustive protocol (0 h), and 0.5, 1, 2, 3 and 5 h following the exhaustive protocol. All measurements were derived from the average of two MO_2_ cycles, except for the 0 h value, which was taken during the first measurement immediately after the exhaustive protocol. Additionally, peak cardiorespiratory responses (i.e. the highest arterial–venous O_2_ content difference, cardiac output, stroke volume and heart rate measured at any time point throughout the recovery period independently from MO_2max_) and time to peak cardiorespiratory responses (i.e. the time elapsed from the beginning of the cardiorespiratory measurements following exhaustive exercise to the peak responses) were determined for each fish.

The relative ventricular mass was calculated as wet mass of the ventricle/body mass × 100. To determine the relative per cent of ventricular compact myocardium, the spongy and compact layers were separated, dried and weighed following the methods of Farrell *et al*. [35]⁠. The percentage compact myocardium was calculated as dry mass of compact myocardium/dry mass of ventricle × 100. The relative spleen mass was calculated as wet mass of the spleen/body mass × 100.

### (e) Statistical analyses

All statistical analyses were performed using SPSS Statistics 24 for Windows (IBM Corporation, Armonk, NY, USA). Statistical significance was accepted at *p* < 0.05 and all data are presented as means ± s.e. Differences in biometric and haematological variables were analysed using one-way ANOVA followed by Tukey *post-hoc* or Kruskal–Wallis *H*-test for non-normally distributed data followed by pairwise comparisons using Dunn’s procedure with Bonferroni correction. If variances were heterogeneous, a one-way Welch ANOVA was performed. One-way analyses of covariance (ANCOVAs) on variables where body mass had a significant effect (i.e. resting, maximum and scope for MO_2_, cardiac output and stroke volume, maximum heart rate, ventricular mass and EPOC) were carried out using body mass as a covariate and were thus standardized to an average sized fish of 974–978 g. Body mass was included as a covariate for all other variables but was subsequently removed as no significant effects were found and an ANOVA or Kruskal–Wallis *H*-test was performed instead. Resting and maximum cardiac output were transformed to their natural logarithms to comply with the assumption of homogeneity of variance of the residuals and normality of the residuals, respectively, while cardiac output scope was square root transformed to comply with the assumption of normality of the residuals. Resting heart rate was transformed to its natural logarithm to comply with the assumption of normality. Outliers were maintained in the analyses if removing or including them did not significantly affect the statistical outcome. Cardiorespiratory dynamics following the exhaustive exercise protocol were analysed using a linear mixed model with fish individual as subject variable and the fixed factors being time (pre-exhaustion, immediately post-exhaustion and 0.5, 1, 2, 3 and 5 h after the exhaustion protocol), treatment (normoxia, hyperoxia_acute_ and hyperoxia_chronic_) and the interaction between time and treatment. For MO_2_, cardiac output and stroke volume, body mass was included as a covariate, while for heart rate and arterial–venous O_2_ content difference, body mass was included as a covariate and subsequently removed as there were no significant effects. The covariance structure was either first-order autoregressive (AR1) or heterogeneous autoregressive, depending on which provided the best fit to the models as indicated by the lowest Akaike’s information criterion (AIC). MO_2_ and arterial–venous O_2_ differences were transformed to their natural logarithm to comply with the assumption of homoscedasticity of the residuals. If significant interactions between time and treatment were found, these were further explored with among- and within-treatments pairwise comparisons, where Bonferroni correction was applied to adjust for multiple testing. Correlation between ventricular mass and maximum stroke volume across treatments adjusting for body mass was analysed using Pearson’s partial correlations (*R*_Partial_).

## 3. Results

**Figure 1.**
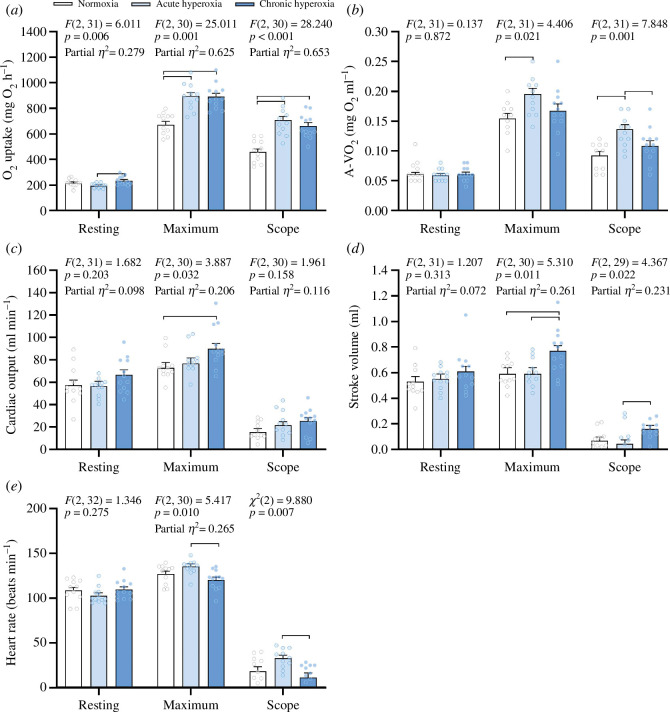
Cardiorespiratory function of yellowtail kingfish (*S. lalandi*) acclimated to normoxia, acutely exposed to hyperoxia (hyperoxia_acute_) or acclimated for a minimum of 3 weeks to hyperoxia (hyperoxia_chronic_) following an exhaustive protocol to elicit maximum cardiorespiratory responses. (*a*) Whole-animal O_2_ uptake rate (MO_2_), (*b*) arterial–venous O_2_ content difference (A-VO_2_), (*c*) cardiac output, (*d*) stroke volume and (*e*) heart rate of yellowtail kingfish in normoxia (*N* = 11), hyperoxia_acute_ (*N* = 11–12) and hyperoxia_chronic_ (*N* = 12). All values are means ± s.e.m. Mass-dependent variables (MO_2_, cardiac output, stroke volume and maximum heart rate) are standardized to an 973.8–978.1 g fish using body mass as a covariate. Main effect from the one-way ANOVA, ANCOVA or Kruskal–Wallis *H*-test are displayed in the figures. For ANCOVA, partial *η*^2^ is provided as an estimate of treatment effect size. Horizontal bars indicate significant differences (*p* < 0.05) between treatments.

Values of *p* are from pairwise comparisons unless otherwise stated. There were no significant differences in body mass or length among the normoxia (1003.6 ± 42.9 g and 40.5 ± 0.6 cm), hyperoxia_acute_ (959.1 ± 41.1 g and 40.3 ± 0.5 cm) and hyperoxia_chronic_ (973.8 ± 41.1 g and 40.0 ± 0.5 cm) treatment groups. SMR differed among treatment groups, with hyperoxia_acute_ having a lower SMR compared with hyperoxia_chronic_ (195.7 ± 8.1 versus 235.1 ± 8.0 mg O_2_ kg^−1^ h^−1^; *p* = 0.005), while SMR in normoxia was intermediate and did not differ significantly from either hyperoxia treatment (216.7 ± 8.4 mg O_2_ kg^−1^ h^−1^; figure 1*a*). Nevertheless, these differences were not accompanied by significant differences in resting arterial–venous O_2_ content difference (normoxia: 0.062 ± 0.003 mg O_2_ ml^−1^; hyperoxia_acute_: 0.060 ± 0.003 mg O_2_ ml^−1^; hyperoxia_chronic_: 0.061 ± 0.003 mg O_2_ ml^−1^; figure 1*b*), cardiac output (normoxia: 57.3 ± 4.6 ml min^−1^ kg^−1^; hyperoxia_acute_: 56.4 ± 4.4 ml min^−1^ kg^−1^; hyperoxia_chronic_: 66.7 ± 4.4 ml min^−1^ kg^−1^; figure 1*c*), stroke volume (normoxia: 0.53 ± 0.04 ml kg^−1^; hyperoxia_acute_: 0.55 ± 0.04 ml kg^−1^; hyperoxia_chronic_: 0.61 ± 0.04 ml kg^−1^; figure 1*d*) or heart rate (normoxia: 108.7 ± 3.2 beats min^−1^; hyperoxia_acute_: 102.8 ± 3.1 beats min^−1^; hyperoxia_chronic_: 109.5 ± 3.1 beats min^−1^; figure 1*e*).

**Figure 2.**
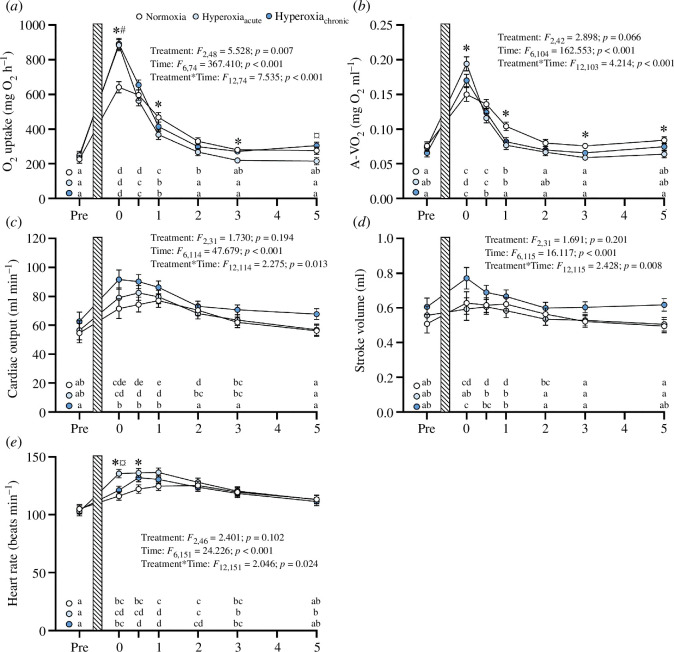
Cardiorespiratory dynamics of yellowtail kingfish (*S. lalandi*) acclimated to normoxia, acutely exposed to hyperoxia (hyperoxia_acute_) or acclimated for a minimum of 3 weeks to hyperoxia (hyperoxia_chronic_) following an exhaustive exercise protocol to elicit maximum cardiorespiratory responses. (*a*) Whole-animal O_2_ uptake rate (MO_2_), (*b*) arterial–venous O_2_ content difference (A-VO_2_), (*c*) cardiac output, (*d*) stroke volume and (*e*) heart rate of yellowtail kingfish in normoxia (*N* = 11), hyperoxia_acute_ (*N* = 11) and hyperoxia_chronic_ (*N* = 12). All values are means ± s.e.m. Mass-dependent variables (MO_2_, cardiac output and stroke volume) are standardized to a 973.8 g fish using body mass as a covariate. Pre-stands for pre-exhaustion values. The hatched bar represents the 3 min exhaustive protocol. The main effects of the linear mixed model are displayed in the figures. Dissimilar letters indicate general statistically significant (*p* < 0.05) differences among sampling times within treatments. * denotes significant differences at a given sampling point between normoxia and hyperoxia_acute_, # denotes significant differences between normoxia and hyperoxia_chronic_ and ¤ denotes significant differences between hyperoxia_acute_ and hyperoxia_chronic_.

Following exhaustive exercise, fish in both acute and chronic hyperoxia had a 33% larger MO_2max_ (*p* < 0.001 in both cases) compared with fish in normoxia (normoxia: 672.9 ± 25.4 mg O_2_ kg^−1^ h^−1^; hyperoxia_acute_: 898.6 ± 25.8 mg O_2_ kg^−1^ h^−1^; hyperoxia_chronic_: 892.1 ± 24.5 mg O_2_ kg^−1^ h^−1^; figure 1*a*) and reached MO_2max_ following exercise faster than normoxia (*p* < 0.001 in both cases, table 1). These differences in MO_2_, however, had disappeared 30 min after the exhaustive exercise protocol (figure 2*a*). Despite the larger MO_2_ during the start of the recovery period in hyperoxia, there were no significant differences in EPOC, EPOC duration or the rate of EPOC repayment among treatments (table 1). Still, the elevated MO_2max_ in hyperoxia resulted in aerobic scope being elevated by 54% in the hyperoxia_acute_ treatment (710.3 ± 26.2 mg O_2_ kg^−1^ h^−1^; *p* < 0.001) and by 43% in the hyperoxia_chronic_ treatment (663.8 ± 24.9 mg O_2_ kg^−1^ h^−1^; *p* < 0.001) compared with normoxia (462.6 ± 26.2 mg O_2_ kg^−1^ h^−1^; figure 1*a*). Nevertheless, the mechanisms by which the elevated MO_2max_ was achieved under hyperoxia differed markedly among treatments.

**Table 1.** Peak cardiorespiratory responses and excess post-exercise O_2_ consumption (EPOC) of yellowtail kingfish in normoxia, acutely transferred to hyperoxia and acclimated to hyperoxia.

variable	normoxia	hyperoxia_acute_	hyperoxia_chronic_	statistics
‍time to MO_2max_ (h)	0.26 ± 0.09b	0.01 ± 0.01a	0.02 ± 0.02a	*χ*^2^(2) = 16.017, *p* < 0.001
‍peak A-VO_2_ (mg O_2_ ml^−1^)	0.17 ± 0.01	0.20 ± 0.01	0.17 ± 0.01	*F*(2,31) = 2.011, *p* = 0.151
‍time to peak A-VO_2_ (h)	0.11 ± 0.02b	0.01 ± 0.01a	0.02 ± 0.01a	*χ*^2^(2) = 21.380, *p* < 0.001
peak cardiac output (ml min^−1^)	81.5 ± 5.0	89.1 ± 5.0	98.4 ± 4.8	*F*(2,30) = 2.976, *p* = 0.066, partial *η*^2^ = 0.166
time to peak cardiac output (h)	0.87 ± 0.18b	0.47 ± 0.09ab	0.31 ± 0.08a	Welch’s *F*(2, 19.112) = 4.320, *p* = 0.028
peak stroke volume (ml)	0.69 ± 0.5	0.67 ± 0.05	0.80 ± 0.05	*F*(2,30) = 2.312, *p* = 0.116, partial *η*^2^ = 0.134
time to peak stroke volume (h)	0.63 ± 0.15b	0.41 ± 0.10ab	0.13 ± 0.04a	*χ*^2^(2) = 8.408, *p* = 0.015
peak heart rate (beats min^−1^)	142.1 ± 3.1	145.3 ± 6.2	145.8 ± 8.8	*F*(2,31) = 0.589, *p* = 0.561
‍time to peak heart rate (h)	1.86 ± 0.47b	0.48 ± 0.23a	0.45 ± 0.11a	*χ*^2^(2) = 9.761, *p* = 0.008
EPOC (mg O_2_)	486.4 ± 41.9	428.0 ± 39.5	469.4 ± 37.6	*F*(2, 26) = 0.555, *p* = 0.581, partial *η*^2^ = 0.041
EPOC duration (h)	3.33 ± 0.26	2.52 ± 0.29	2.95 ± 0.27	*F*(2, 27) = 2.056, *p* = 0.148
EPOC/time (mg O_2_ h^−1^)	157.9 ± 17.2	173.5 ± 16.2	166.5 ± 15.4	*F*(2, 26) = 0.217, *p* = 0.806, partial *η*^2^ = 0.016

Peak and time to peak O_2_ uptake (MO_2_), arterial–venous O_2_ content difference (A-VO_2_), cardiac output, stroke volume, heart rate, EPOC, EPOC duration and rate of EPOC repayment (EPOC/time) of yellowtail kingfish (*S. lalandi*) acclimated to normoxia, acutely exposed to hyperoxia or acclimated for a minimum of 3 weeks to hyperoxia following an exhaustive protocol to elicit maximum cardiorespiratory responses. Sample sizes for peak and time to peak cardiorespiratory responses are *n* = 11, 11 and 12 for normoxia, hyperoxia_acute_ and hyperoxia_chronic_, respectively, while for EPOC and related parameters are *n* = 9, 10 and 11, respectively. All values are means ± s.e.m. Mass-dependent variables (cardiac output, stroke volume, EPOC and EPOC/time) are standardized to a 977.5–973.8 g fish using body mass as a covariate. Results from the one-way ANOVA or ANCOVA are displayed under statistics. For ANCOVA, partial *η*^2^ is provided as an estimate of effect size of treatment. Dissimilar letters indicate statistically significant (*p* < 0.05) differences between treatments.

The higher post-exhaustive exercise aerobic metabolism in hyperoxia_acute_ compared with normoxia was mainly driven by an increased maximum arterial–venous O_2_ content difference (0.196 ± 0.01 versus 0.155 ± 0.01 mg O_2_ ml^−1^; *p* = 0.018); a difference that was most evident immediately after exhaustive exercise (i.e. at 0 h post-stress; figure 2*b*), while maximum arterial–venous O_2_ difference in the hyperoxia_chronic_ treatment (0.168 ± 0.01 mg O_2_ ml^−1^) did not differ significantly from the other treatments (figure 1*b*). Consequently, the scope for arterial–venous O_2_ content difference was larger in hyperoxia_acute_ (0.138 ± 0.008 mg ml^−1^) than in normoxia (0.092 ± 0.008 mg ml^−1^; *p* = 0.002) and hyperoxia_chronic_ (0.108 ± 0.008 mg ml^−1^; *p* = 0.039; figure 1*b*). None of the peak cardiorespiratory responses differed between normoxia and hyperoxia_acute_ (table 1). Still, all of the peaks in cardiorespiratory responses following exercise occurred quicker in the hyperoxia_acute_ compared with normoxia (*p* < 0.001) with the exception of cardiac output and stroke volume where no significant differences in time to peak responses were found (table 1).

In contrast to the hyperoxia_acute_ treatment where the larger MO_2max_ was primarily explained by higher arterial–venous O_2_ content, the enhanced MO_2max_ observed in hyperoxia_chronic_ was achieved via an increased maximum cardiac output (89.8 ± 4.7 ml min^−1^ kg^−1^), which was significantly larger than in normoxia (72.8 ± 4.9 ml min^−1^ kg^−1^, *p* = 0.037), but not relative to hyperoxia_acute_ (76.9 ± 4.9 ml min^−1^ kg^−1^; *p* = 0.173; figure 1*c*). Still, cardiac output scope did not differ among treatments (normoxia: 15.4 ± 3.2 ml min^−1^ kg^−1^; hyperoxia_acute_: 21.6 ± 3.1 ml min^−1^ kg^−1^; hyperoxia_chronic_: 25.2 ± 3.1 ml min^−1^ kg^−1^; figure 1*c*). The greater maximum cardiac output in hyperoxia_chronic_ was mainly driven by a greater stroke volume compared with normoxia (0.77 ± 0.04 versus 0.59 ± 0.05 ml kg^−1^; *p* = 0.026) and hyperoxia_acute_ (0.59 ± 0.05 ml kg^−1^; *p* = 0.028; figure 1*d*). As a result, the stroke volume scope was significantly greater in hyperoxia_chronic_ (0.158 ± 0.029 ml kg^−1^) compared with hyperoxia_acute_ (0.045 ± 0.029 ml kg^−1^; *p* = 0.029), and showed a similar trend of being larger compared with normoxia (0.067 ± 0.029 ml kg^−1^; *p* = 0.099; figure 1*d*). Moreover, the maximum heart rate was higher in hyperoxia_acute_ (135.2 ± 3.3 beats min^−1^ standardized to a 973.8 g fish) compared with hyperoxia_chronic_ (120.4 ± 3.1 beats min^−1^ standardized to a 973.8 g fish; *p* = 0.008), while neither of the hyperoxia treatments differed significantly from normoxia (127.0 ± 3.3 beats min^−1^ standardized to a 973.8 g fish; figure 1*e*). As a result, the heart rate scope in hyperoxia_acute_ was significantly larger than in hyperoxia_chronic_ (33.0 ± 3.3 beats min^−1^ versus 11.3 ± 5.1 beats min^−1^; *p* = 0.006), but not compared with normoxia (18.2 ± 5.2 beats min^−1^; *p* = 0.133; figure 1*e*). None of the peak cardiorespiratory responses differed among treatments (table 1), yet, all of the peaks in cardiorespiratory responses following exercise occurred quicker in hyperoxia_chronic_ compared with normoxia (e.g. time to MO_2max_, peak arterial–venous O_2_ content difference and heart rate, *p* < 0.001). While cardiac output and stroke volume peaked faster in hyperoxia_chronic_ compared with normoxia (*p* = 0.027 and *p* = 0.016, respectively), hyperoxia_acute_ did not differ significantly from either treatment (table 1).

**Figure 3.**
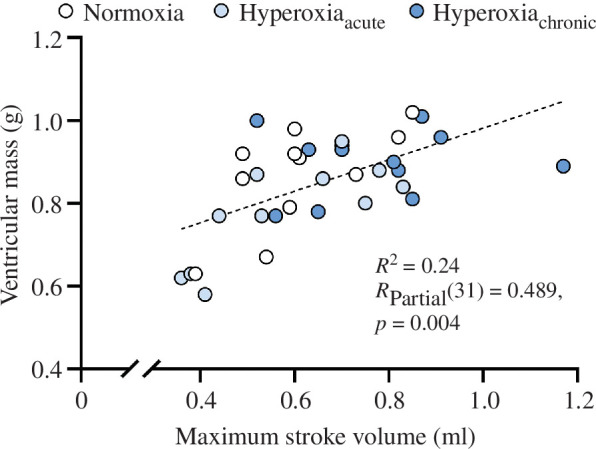
Weight-controlled relationship between ventricular mass and maximum stroke volume following exercise in yellowtail kingfish (*S. lalandi*) acclimated to normoxia, acutely exposed to hyperoxia (hyperoxia_acute_) or acclimated for a minimum of 3 weeks to hyperoxia (hyperoxia_chronic_). Linear regression between ventricular mass and maximum stroke volume across exposure treatments is indicated by a hatched line (*N* = 34). Pearson’s partial correlation (*R*_Partial_) indicates the strength and direction of the association between the two variables. The *p*-value indicates statistically significant correlations (*p* < 0.05) between ventricular mass and maximum stroke volume across treatments.

Ventricular mass differed among treatments when standardized using mass as a covariate, with hyperoxia_chronic_ having a significantly larger ventricle than normoxia (*p* = 0.017) and hyperoxia_acute_ (*p* < 0.001), while hyperoxia_acute_ trended towards a smaller ventricle compared with normoxia (*p* = 0.104, table 2). Consistent with the elevated maximum stroke volume in hyperoxia_chronic_, the relative ventricular mass was ~8% larger in hyperoxia_chronic_ compared with normoxia (*p* = 0.013), and ~12% larger compared with the hyperoxia_acute_ treatment (*p* < 0.001, table 2). Moreover, bivariate Pearson’s correlation indicated a significant linear relationship between ventricular mass and maximum stroke volume when analysed across treatment groups (*R*_Pearson_(32) = 0.586, *p* < 0.001). When adjusting for body mass, Pearson’s partial correlation showed that the relationship remained significant (figure 3). The relative proportion of compact myocardium was significantly greater in hyperoxia_acute_ compared with hyperoxia_chronic_ (*p* = 0.019), a trend that was also somewhat evident when compared with normoxia (*p* = 0.072; see table 2). No other measured morphological or haematological features of kingfish differed among treatments (table 2).

**Table 2.** Morphological and haematological properties of yellowtail kingfish (*S. lalandi*) normoxia, acutely transferred to hyperoxia and acclimated to hyperoxia.

variable	normoxia	hyperoxia_acute_	hyperoxia_chronic_	statistics
ventricular mass (g)	0.85 ± 0.01a	0.81 ± 0.01a	0.90 ± 0.01b	*F*(2,34) = 14.203, *p* < 0.001 partial *η*^2^ = 0.480
relative ventricular mass (%)	0.086 ± 0.001a	0.083 ± 0.001a	0.093 ± 0.001b	*F*(2, 32) = 11.274, *p* = 0.001
proportion compact myocardium (%)	23.7 ± 0.8ab	25.8 ± 0.5b	23.2 ± 0.6a	*F*(2, 32) = 4.622, *p* = 0.017
relative spleen mass (%)	0.075 ± 0.004	0.074 ± 0.004	0.077 ± 0.004	*F*(2, 32) = 0.231, *p* = 0.795
haematocrit (%)	40.5 ± 0.9	40.8 ± 0.7	40.1 ± 1.1	*F*(2, 32) = 0.084, *p* = 0.920
haemoglobin (g l^−1^)	114.8 ± 1.5	116.6 ± 2.0	112.1 ± 2.7	*F*(2, 32) = 0.509, *p* = 0.606
MCHC (g l^−1^)	284.1 ± 4.5	286.2 ± 4.0	280.4 ± 6.2	*F*(2, 32) = 0.149, *p* = 0.862
pH	7.16 ± 0.05	7.10 ± 0.03	7.09 ± 0.02	Welch’s *F*(2, 19.249) = 0.906, *p* = 0.421

Yellowtail kingfish (*S. lalandi*) acclimated to normoxia (*n* = 11), acutely exposed to hyperoxia (hyperoxia_acute_, *n* = 11–12) or acclimated for a minimum of 3 weeks to hyperoxia (hyperoxia_chronic_, *n* = 12). MCHC: mean corpuscular haemoglobin concentration. All values are means ± s.e.m. Results from the one-way ANOVA/ANCOVA or Welch ANOVA are displayed under statistics. Ventricular mass was standardized to a 978.1 g fish using body mass as a covariate (ANCOVA). Partial *η*^2^ is provided as an estimate of effect size of treatment. Dissimilar letters indicate statistically significant (*p* < 0.05) differences between treatments.

## 4. Discussion

Kingfish in hyperoxia exhibited MO_2max_-driven expansions of aerobic scope. However, the mechanisms whereby this was achieved starkly contrasted depending on whether the fish were acutely exposed to hyperoxia (∼20 h of exposure) or chronically acclimated for 3–5 weeks. As far as we are aware, this is the first study analysing and comparing the underlying cardiorespiratory mechanisms of the enhanced metabolic performance of fish exposed to different durations of environmental hyperoxia.

### (a) Acute hyperoxia increases metabolic performance via increases in tissue O_2_ extraction

MO_2max_ of kingfish acutely exposed to hyperoxia was 33% larger than in normoxia. Similar responses to acute hyperoxia exposure have been observed in other fish species such as rainbow trout [9,10]⁠, European perch [11]⁠, common galaxias [14]⁠ and two triplefin species [13]⁠. Furthermore, the previous studies on rainbow trout suggest that elevations in MO_2max_ are facilitated mainly by increases in maximum cardiac output [9]⁠ or a combination of elevations in maximum cardiac output and arterial–venous O_2_ content difference [10]⁠. Here, in acutely hyperoxia-exposed kingfish, the response was somewhat different from these earlier studies as the larger MO_2max_ was mainly owing to a ∼26% larger arterial–venous O_2_ content difference. Previous studies have reported large drops in PaO_2_ in fish exercised in normoxia, possibly resulting from insufficient gill ventilation and/or a gill O_2_ diffusion limitation in normoxia [10,18,36,37]⁠. Thus, an important mechanism that allows for an increased arterial–venous O_2_ content difference in acutely transferred fish is likely a protective effect of hyperoxia on arterial oxygenation immediately following exercise, which allows for arterial blood to remain close to full saturation [10]⁠. Still, studies in perch and trout indicate that cardiac function can be expected to improve in hyperoxia via a higher O_2_ delivery to the spongy myocardium [9,15]⁠, as well as to the compact myocardium when coronary PaO_2_ increases [10]⁠. Yet, this potential effect was not immediately obvious in the current study as the maximum cardiac output of the hyperoxia_acute_ group did not differ significantly from kingfish in normoxia. Still, the studies on trout have yielded somewhat conflicting results regarding the effects of acute hyperoxia on cardiac function [9,10]⁠. When trout in hyperoxia had an elevated arterial–venous O_2_ content difference compared with normoxia, maximum stroke volume did not differ between treatments and the higher cardiac output was mainly driven by an increased heart rate [10]⁠. On the other hand, when arterial–venous O_2_ content difference was similar to trout in normoxia, the larger cardiac output in hyperoxia was stroke volume driven [9]⁠. It is therefore possible that when tissue O_2_ extraction was exacerbated, reductions in the O_2_ levels of venous blood returning to the heart nullified the benefits of hyperoxia on cardiac contractility. In fact, peak stroke volume in hyperoxia_acute_ was 14% larger than stroke volume occurring at MO_2max_ suggesting that stroke volume cannot be maximized at the time when MO_2_ and arterial–venous O_2_ content difference is largest. It is noteworthy that heart rate was also elevated immediately following exhaustion in hyperoxia_acute_ compared with the other treatments (figure 2*e*), therefore, it is also possible that the elevated heart rate prevented stroke volume from being maximized by reducing diastolic filling time and contractile force (for reviews on the negative force–frequency relationship and Frank-Starling effects, e.g. [38–40]⁠).

### (b) Maintenance of improved metabolic performance in kingfish chronically acclimated to hyperoxia is achieved via increased cardiac performance

Following long-term acclimation to hyperoxia, the chief factor allowing for an enhanced metabolic performance shifted to increased maximum cardiac output. Indeed, the elevated maximum cardiac output in hyperoxia_chronic_ was mainly driven by an increased stroke volume, while heart rate responses to exhaustive exercise did not differ from kingfish in normoxia. This response resembles the positive inotropic cardiac effects previously observed in perch and rainbow trout acutely transferred to hyperoxia [9,15,16]⁠. Nevertheless, if an enhanced O_2_ delivery to the heart was the only underlying mechanism, it would be expected that, like in perch and trout, kingfish acutely exposed to hyperoxia would experience similar increases in maximum cardiac output as chronically acclimated ones. This was not the case as the maximum cardiac output of the hyperoxia_acute_ group was intermediate between normoxia and hyperoxia_chronic_ treatment groups and did not differ significantly from either treatment.

Swimming fish increase cardiac output via increases of either stroke volume, heart rate or combinations thereof [41]⁠. Even so, it appears that kingfish maximally exercised in normoxia have a limited ability to increase heart rate or stroke volume and thus rely on large increases in tissue O_2_ extraction [21,22]⁠. This inability to increase heart rate and stroke volume may stem from the high resting heart rate, which leaves little scope for further elevations in heart rate and may hinder elevations in stroke volume [38–40]⁠. Like kingfish in normoxia, it could be hypothesized that despite the added O_2_ supply to the heart, there was little margin to increase stroke volume further upon acute exposure to hyperoxia. Thus, it appears that for this species to benefit fully from the added myocardial O_2_ supply from environmental hyperoxia and further increase maximum stroke volume, plastic changes to the morphology and composition of the heart ventricle are required as suggested by the larger relative ventricular mass in chronically hyperoxia-acclimated kingfish. Indeed, a bigger ventricle should allow for a larger stroke volume [42,43]⁠, a relationship highlighted here by the positive correlation between ventricular mass and stroke volume in kingfish across treatments (figure 3). Perhaps surprisingly, the proportion of compact myocardium between normoxia and hyperoxia_chronic_ did not differ, while it was significantly elevated in hyperoxia_acute_ compared with hyperoxia_chronic_, likely owing to a reduction in spongy myocardium rather than an actual growth of the compact myocardium given the trend towards a reduced ventricular mass in the acute treatment. Still, the similar proportions of compact myocardium between fish in normoxia and chronically acclimated to hyperoxia suggest that long-term cardiac growth in hyperoxia occurred homogeneously across myocardial layers. It is unclear which mechanisms promote ventricular growth in kingfish acclimated to hyperoxia, although it does not appear to be a general feature in fish, as common galaxias acclimated to hyperoxia for 5 months displayed no changes in relative ventricular mass [14]⁠.

Despite the significant cardiac growth in hyperoxia_chronic_ compared with normoxia, this phenomenon only partially explained the significant differences in maximum stroke volume (i.e. stroke volume occurring at MO_2max_). While maximum stroke volume in hyperoxia_chronic_ was ∼31% larger than in normoxia, the difference in peak stroke volume was only ∼16%, since the difference between maximum stroke volume and peak stroke volume in normoxia was larger than in hyperoxia_chronic_. It is likely that the larger differences between peak stroke volume and maximum stroke volume in normoxia were owing to insufficient cardiac oxygenation at MO_2max_, when tissue O_2_ extraction was elevated. Similarly, trout acutely transferred to hyperoxia that sustain a larger MO_2max_ via increases in cardiac output while maintaining a similar arterial–venous O_2_ content difference compared with normoxia, also display a somewhat elevated stroke volume, which coincides with a significantly larger PvO_2_ [9]. Therefore, it is likely that the larger maximum cardiac output observed in hyperoxia_chronic_ was driven by a combination of cardiac growth and improved cardiac oxygenation. This hypothesis is also supported by the delayed peak in stroke volume observed in normoxia, which occurred 0.5 h after stroke volume had peaked in hyperoxia_chronic_, probably as the amounts of O_2_ received by the spongy myocardium during periods of exacerbated tissue O_2_ extraction were insufficient. Thus, the mechanisms that enable hyperoxia_chronic_ kingfish to sustain a higher cardiac output at MO_2max_ compared with normoxia, which includes ventricular growth, also allow them to maximize cardiac function almost concurrently when tissue O_2_ extraction is maximized.

### (c) Shift from enhanced tissue O_2_ extraction to improved cardiac performance with prolonged hyperoxia acclimation—side effect or adaptive response?

Increasing tissue O_2_ delivery through elevations in blood flow rather than tissue O_2_ extraction may have certain physiological advantages on top of a potentially increased venous O_2_ reserve during metabolically active events to further draw upon if needed. For example, an enhanced O_2_ extraction by metabolically active tissues is partly reliant on regional rightward and downward shifts of the O_2_ dissociation curve (Bohr and Root effects, respectively [44,45]⁠), which partly result from acidosis mainly resulting from the exacerbated hydrolysis of ATP and accumulation of protons as they cannot be used in oxidative phosphorylation owing to lack of O_2_ [46]⁠. Exacerbated acidosis has detrimental effects including negative contractile effects on the cardiac and skeletal muscle of some fish species [47,48]⁠ and has been proposed as a potential cause for post-exercise mortality [49]⁠. Bearing this in mind, it may be hypothesized that an improved capacity to deliver blood, as indicated by the higher maximum cardiac output in chronically hyperoxia-exposed kingfish, allows for a better supply of metabolically active tissues with O_2_ and more efficient removal of metabolic by-products, while requiring less dramatic regional acid–base disturbances. This would facilitate maintaining homeostasis and possibly even optimizing other performance aspects including aerobic swimming capacity.

On the other hand, it is possible that at very high tissue O_2_ levels, aerobic mitochondrial respiration is limited by some metabolic substrate other than O_2_ and therefore tissues do not maximize extraction, as the volume of oxygenated blood that can be delivered to the tissues (owing to higher maximum cardiac output) is elevated. This hypothesis may be tested by examining whether even higher levels of hyperoxia allow for larger MO_2max_, or whether MO_2max_ indeed reaches an upper plateau. Another contributing factor to the lower maximum tissue O_2_ extraction in hyperoxia_chronic_ so that it no longer differs from normoxia may be a downregulation of biochemical processes that allow for enhanced O_2_ extraction under chronic hyperoxia, e.g. reduced abundance or activity of plasma-accessible carbonic anhydrases or remodelling of gill morphology [50,51]⁠. For example, goldfish acclimated for 2 weeks to hyperoxia (>320% air saturation) at 25°C displayed a 31.7% increase in interlamellar cell mass compared with fish in normoxia [52]⁠. If a similar interlamellar proliferation occurred in kingfish in hyperoxia, it could hinder O_2_ exchange at the gills, a reduction in extractive capabilities that would be then offset by an elevated maximum cardiac output, resulting in a similar MO_2max_ as in hyperoxia_acute_.

## 5. Conclusions and future perspectives

We show for the first time some of the mechanisms that allow for an elevated aerobic capacity in fish chronically exposed to hyperoxia. Our results suggest that the mechanisms that allow for an elevated MO_2max_ in hyperoxia changes dynamically as hyperoxia exposure is sustained over time. Acutely exposed fish elevate MO_2max_ via increased tissue extraction while chronically acclimated fish sustain the elevated metabolic performance through increased maximum cardiac output and stroke volume, facilitated by cardiac growth resulting in increased relative ventricular mass. Based on our results, at 200% air saturation, kingfish suffer no obvious negative consequences from hyperoxia compared with normoxia and thus may be comfortably used in situations resulting in stress (e.g. crowding, transport, grading) to protect their cardiorespiratory function. Similarly, there is recent evidence that O_2_ supplementation extends the reproductive window of some species [53]⁠, slightly increases upper thermal tolerance [1,11,15,16]⁠ and may improve food intake at supra-optimal temperatures [54]⁠. It is at these higher temperatures that the benefits of an enhanced aerobic scope may be particularly manifested for both wild and farmed fish as average water temperatures as well as frequency and intensity of heatwaves increase.

## Data Availability

Data supporting this study is available on Dryad [55].
